# Supplementary material for: Morphological variation associated with trophic niche expansion within a lake population of a benthic fish
Source: PLoS One. 2020 Apr 23;15(4):e0232114. doi: 10.1371/journal.pone.0232114 (PMC7179883; doi:10.1371/journal.pone.0232114)
Supplement: S1 Table — (DOCX) [file pone.0232114.s001.docx]

**S1 Table.** **Results of the principal component analysis (PCA) for standardized morphological trait values.**

|  | PC1 | PC2 | PC3 | PC4 | PC5 |
| --- | --- | --- | --- | --- | --- |
| Contribution ratio (%) | 93.4 | 3.94 | 1.29 | 0.59 | 0.36 |
| Cumulative contribution ratio (%) | 93.4 | 97.3 | 98.6 | 99.2 | 99.5 |
| Contribution ratio after the exclusion of PC1’s contribution (%) | - | 59.1 | 19.4 | 8.79 | 5.43 |
| Eigenvalue | 7.47 | 0.32 | 0.10 | 0.05 | 0.03 |
| Standard length (SL) | -0.99 | -0.13 | 0.03 | -0.03 | 0.03 |
| Body depth (BD) | -0.97 | -0.16 | -0.06 | 0.07 | -0.09 |
| Body width (BW) | -0.99 | -0.10 | -0.04 | 0.02 | -0.08 |
| Caudal peduncle depth (CPD) | -0.97 | -0.19 | -0.02 | 0.09 | 0.12 |
| Head length (HL) | -0.99 | -0.06 | 0.06 | -0.08 | 0.01 |
| Snout length (SnL) | -0.99 | 0.02 | 0.05 | -0.14 | -0.01 |
| Mouth length (ML) | -0.92 | 0.33 | 0.20 | 0.09 | -0.01 |
| Mouth width (MW) | -0.91 | 0.34 | -0.23 | -0.01 | 0.02 |
