# Supplementary material for: Morphological variation associated with trophic niche expansion within a lake population of a benthic fish
Source: PLoS One. 2020 Apr 23;15(4):e0232114. doi: 10.1371/journal.pone.0232114 (PMC7179883; doi:10.1371/journal.pone.0232114)
Supplement: S2 Table — (DOCX) [file pone.0232114.s002.docx]

**S2 Table.** **Primer sequences and the related information of microsatellite primers for *Pseudogobio esocinus*.**

| Locus | Primer sequence | | Repeat motif | | | Fluorescence | Size range |
| --- | --- | --- | --- | --- | --- | --- | --- |
| Pes1_07 | F: | CAGGACCAGGCTACCGTGACGCCCTGATGCCAACATT | | (TG)_15_ | NED | | 115–143 |
|  | R: | GTTTCTTGGCAGACAGCCCACTCTGTT | |  |  | |  |
| Pes1_09 | F: | CAGGACCAGGCTACCGTGTGATGTGATGCGAGTTACAGGC | | (TG)_10_ | NED | | 286–308 |
|  | R: | GTTTCTTTGCTGTTTACAATGCCAGGGT | |  |  | |  |
| Pes1_12 | F: | CGGAGAGCCGAGAGGTGAGAAAGAAGATGCTCTTTGCTTCCA | | (AC)_13_ | PET | | 304–330 |
|  | R: | GTTTCTTGATTAAATCTTGTCACACGCTGGTA | |  |  | |  |
| Pes1_13 | F: | GCCTCCCTCGCGCCACCGTGTTTAGGTCTTGCAATGG | | (TG)_13_ | FAM | | 111–165 |
|  | R: | GTTTCTTAAGCTGCTGTCACTGCCGTA | |  |  | |  |
| Pes1_14 | F: | GCCTCCCTCGCGCCACATACAGGCTCTTCCCAGCG | | (AC)_13_ | FAM | | 195–253 |
|  | R: | GTTTCTTTTTGACCTTTGACCTTCGGG | |  |  | |  |
| Pes1_17 | F: | GCCTTGCCAGCCCGCCAAGCCTGGCACTAAAGAACG | | (AC)_12_ | VIIC | | 236–256 |
|  | R: | GTTTCTTTGCTCTGTCACAGCTGGCTT | |  |  | |  |
| Pes1_18 | F: | GCCTTGCCAGCCCGCTTGCTCAAGAGTATGTACAGCAACG | | (GT)_12_ | VIC | | 373–403 |
|  | R: | GTTTCTTCCGTGAGACATCATCTACACTGC | |  |  | |  |
| Pes1_21 | F: | CAGGACCAGGCTACCGTGTGACTCTCACCTGCACCCG | | (CA)_11_ | NED | | 325–335 |
|  | R: | GTTTCTTTGAGTAGAACGCTCGCATGG | |  |  | |  |
| Pes1_23 | F: | CGGAGAGCCGAGAGGTGTGCACAGTCCAAAGAGACGAA | | (AC)_13_ | PET | | 170–220 |
|  | R: | GTTTCTTTTGCATTCACGTGTTGTGACAG | |  |  | |  |
| Pes1_24 | F: | CGGAGAGCCGAGAGGTGCAGCCCTTGTGCCAGTTCTC | | (AC)_10_ | PET | | 337–347 |
|  | R: | GTTTCTTACTGGAATCACTGCGGCATC | |  |  | |  |
| Pes2_03 | F: | GCCTCCCTCGCGCCATTGTTAAAGGGTCACTACGTAATCAAG | | (CA)_19_ | FAM | | 239–285 |
|  | R: | GTTTCTTGTAGGGTGGGACACTGCTGC | |  |  | |  |
| Pes2_05 | F: | GCCTTGCCAGCCCGCGTCCGGGCCAGTACGGTAA | | (CA)_14_ | VIC | | 192–212 |
|  | R: | GTTTCTTGGTTTGTTGGGAATGAAGGG | |  |  | |  |
| Pes2_07 | F: | CAGGACCAGGCTACCGTGTGTCCATGTCTTCCTTGGCA | | (GT)_17_ | NED | | 125–155 |
|  | R: | GTTTCTTCCGTTCTGTCCAAACAGCATC | |  |  | |  |
| Pes2_09 | F: | CAGGACCAGGCTACCGTGTTGGATGAGTTAATGGAGAATGTCA | | (AC)_14_ | NED | | 272–330 |
|  | R: | GTTTCTTTTCAGCTCCAGCAGTGAATAATG | |  |  | |  |

One out of four universal tail sequences was added to each forward primer for fluorescent labelling during PCR [1].
