# Supplementary material for: Morphological variation associated with trophic niche expansion within a lake population of a benthic fish
Source: PLoS One. 2020 Apr 23;15(4):e0232114. doi: 10.1371/journal.pone.0232114 (PMC7179883; doi:10.1371/journal.pone.0232114)
Supplement: S3 Table — (DOCX) [file pone.0232114.s003.docx]

**S3 Table.** **The results of GLM analyses on the proportion of each food item in gut contents of *Pseudogobio esocinus* in L4, L6, R1, and R7.**

|  | L4 (n = 30) | | |  | L6 (n = 16) | | |  | R1 (n = 13) | | |  | R7 (n = 9) | | |
| --- | --- | --- | --- | --- | --- | --- | --- | --- | --- | --- | --- | --- | --- | --- | --- |
|  |  |  |  |  |  |  |  |  |  |  |  |  |  |  |  |
| Chironomids | Coef. | t | p |  | Coef. | t | p |  | Coef. | t | p |  | Coef. | t | p |
| PC2 | 0.37 | 1.66 | 0.11 |  | -1.94 | -2.02 | 0.07 |  | 0.64 | 1.76 | 0.12 |  | -7.71 | -1.94 | 0.05 |
| PC3 | 0.31 | 1.27 | 0.22 |  | 0.69 | 0.75 | 0.47 |  | 1.59 | 1.69 | 0.13 |  | -1.66 | -0.58 | 0.56 |
| SL | -0.01 | -1.41 | 0.17 |  | -0.004 | -0.85 | 0.41 |  | -0.01 | -1.19 | 0.27 |  | -0.01 | -0.18 | 0.86 |
| totalP | 0.02 | 10.37 | **< 0.001** |  | 0.01 | 3.32 | **0.007** |  | 0.004 | 5.65 | **< 0.001** |  | 0.01 | 1.59 | 0.11 |
| Amphipods | Coef. | z | p |  | Coef. | t | p |  |  |  |  |  |  |  |  |
| PC2 | -1.02 | -4.53 | **< 0.001** |  | 1.75 | 0.87 | 0.40 |  |  |  |  |  |  |  |  |
| PC3 | 2.19 | 4.54 | **< 0.001** |  | 0.23 | 0.14 | 0.89 |  |  |  |  |  |  |  |  |
| SL | -0.004 | -0.86 | 0.39 |  | 0.00 | -0.47 | 0.65 |  |  |  |  |  |  |  |  |
| totalP | 0.03 | 8.96 | **< 0.001** |  | 0.01 | 3.82 | **0.003** |  |  |  |  |  |  |  |  |
| Oligochaetes | Coef. | z | p |  |  |  |  |  |  |  |  |  |  |  |  |
| PC2 | 1.12 | 2.09 | **0.04** |  |  |  |  |  |  |  |  |  |  |  |  |
| PC3 | 1.39 | 2.27 | **0.02** |  |  |  |  |  |  |  |  |  |  |  |  |
| SL | 0.04 | 4.79 | **< 0.001** |  |  |  |  |  |  |  |  |  |  |  |  |
| totalP | 0.01 | 2.28 | **0.02** |  |  |  |  |  |  |  |  |  |  |  |  |
| Mayflies |  |  |  |  |  |  |  |  | Coef. | t | p |  |  |  |  |
| PC2 |  |  |  |  |  |  |  |  | -0.88 | -1.12 | 0.29 |  |  |  |  |
| PC3 |  |  |  |  |  |  |  |  | 0.63 | 0.45 | 0.66 |  |  |  |  |
| SL |  |  |  |  |  |  |  |  | 0.07 | 2.92 | **0.02** |  |  |  |  |
| totalP |  |  |  |  |  |  |  |  | 0.002 | 1.02 | 0.34 |  |  |  |  |
| Caddisflies |  |  |  |  |  |  |  |  | Coef. | t | p |  |  |  |  |
| PC2 |  |  |  |  |  |  |  |  | -0.31 | -0.37 | 0.73 |  |  |  |  |
| PC3 |  |  |  |  |  |  |  |  | 0.01 | 0.01 | 0.99 |  |  |  |  |
| SL |  |  |  |  |  |  |  |  | 0.02 | 0.80 | 0.45 |  |  |  |  |
| totalP |  |  |  |  |  |  |  |  | 5.8E-05 | 0.04 | 0.97 |  |  |  |  |

Explanatory variables for estimation in the models are two principal component scores (PC2, PC3), standard length (SL), and total points of all food items (total P). Coefficients (Coef.) indicate regression estimate values. The t-value and z-value indicate the statistics in quasi- and zero-inflated Poisson models, respectively.
