# Supplementary material for: Morphological variation associated with trophic niche expansion within a lake population of a benthic fish
Source: PLoS One. 2020 Apr 23;15(4):e0232114. doi: 10.1371/journal.pone.0232114 (PMC7179883; doi:10.1371/journal.pone.0232114)
Supplement: S4 Table — (DOCX) [file pone.0232114.s004.docx]

**S4 Table.** **Pairwise-Fst estimated from 14 microsatellite-locus data in Lake Biwa and the surrounding rivers.**

|  | L1 | L2 | L3 | L4 | L5 | L6 | L7 | L8 | L9 | R1 | R2 | R3 | R4 | R5 | R6 | R7 | R8 |
| --- | --- | --- | --- | --- | --- | --- | --- | --- | --- | --- | --- | --- | --- | --- | --- | --- | --- |
| L2 | 0.004 |  |  |  |  |  |  |  |  |  |  |  |  |  |  |  |  |
| L3 | 0.006 | 0.003 |  |  |  |  |  |  |  |  |  |  |  |  |  |  |  |
| L4 | 0.005 | 0.008 | 0.008 |  |  |  |  |  |  |  |  |  |  |  |  |  |  |
| L5 | 0.000 | -0.002 | -0.004 | 0.002 |  |  |  |  |  |  |  |  |  |  |  |  |  |
| L6 | 0.002 | 0.011 | 0.004 | 0.006 | 0.004 |  |  |  |  |  |  |  |  |  |  |  |  |
| L7 | -0.001 | 0.003 | 0.009 | -0.005 | 0.000 | 0.008 |  |  |  |  |  |  |  |  |  |  |  |
| L8 | 0.001 | -0.003 | 0.005 | 0.010 | -0.001 | 0.009 | -0.002 |  |  |  |  |  |  |  |  |  |  |
| L9 | -0.006 | -0.005 | 0.010 | 0.008 | 0.003 | 0.004 | -0.001 | -0.007 |  |  |  |  |  |  |  |  |  |
| R1 | ***0.048*** | ***0.047*** | ***0.063*** | ***0.055*** | ***0.061*** | ***0.069*** | ***0.052*** | ***0.047*** | ***0.037*** |  |  |  |  |  |  |  |  |
| R2 | 0.013 | ***0.028*** | 0.027 | 0.022 | ***0.022*** | 0.024 | ***0.028*** | 0.031 | ***0.024*** | ***0.075*** |  |  |  |  |  |  |  |
| R3 | ***0.067*** | ***0.059*** | ***0.045*** | ***0.056*** | ***0.069*** | ***0.063*** | ***0.056*** | ***0.084*** | ***0.077*** | ***0.114*** | ***0.086*** |  |  |  |  |  |  |
| R4 | ***0.025*** | ***0.035*** | 0.020 | 0.019 | ***0.033*** | 0.015 | 0.024 | 0.033 | ***0.030*** | ***0.084*** | ***0.050*** | ***0.050*** |  |  |  |  |  |
| R5 | ***0.056*** | ***0.071*** | ***0.064*** | ***0.064*** | ***0.088*** | ***0.061*** | ***0.068*** | ***0.083*** | ***0.072*** | ***0.112*** | ***0.096*** | ***0.090*** | 0.029 |  |  |  |  |
| R6 | ***0.043*** | ***0.060*** | ***0.056*** | ***0.040*** | ***0.069*** | ***0.040*** | ***0.045*** | ***0.061*** | ***0.056*** | ***0.098*** | ***0.066*** | ***0.077*** | 0.024 | 0.019 |  |  |  |
| R7 | ***0.051*** | ***0.063*** | ***0.057*** | ***0.053*** | ***0.063*** | ***0.059*** | ***0.054*** | 0.063 | ***0.064*** | ***0.111*** | ***0.085*** | ***0.086*** | ***0.060*** | ***0.082*** | ***0.068*** |  |  |
| R8 | ***0.090*** | ***0.087*** | ***0.086*** | ***0.096*** | ***0.106*** | ***0.086*** | ***0.095*** | ***0.109*** | ***0.103*** | ***0.145*** | ***0.124*** | ***0.091*** | ***0.083*** | ***0.073*** | ***0.080*** | ***0.105*** |  |
| R9 | ***0.305*** | ***0.303*** | ***0.310*** | ***0.286*** | ***0.311*** | ***0.307*** | ***0.286*** | ***0.295*** | ***0.308*** | ***0.329*** | ***0.355*** | ***0.332*** | ***0.274*** | ***0.300*** | ***0.314*** | ***0.301*** | ***0.328*** |

Figures in italics are significant (significance level = 0.05) after Holm’s correction.
