# Supplementary material for: Morphological variation associated with trophic niche expansion within a lake population of a benthic fish
Source: PLoS One. 2020 Apr 23;15(4):e0232114. doi: 10.1371/journal.pone.0232114 (PMC7179883; doi:10.1371/journal.pone.0232114)
Supplement: S5 Table — (DOCX) [file pone.0232114.s005.docx]

**S5 Table.** **Summary of polymorphism of 14 microsatellite-locus of *Pseudogobio esocinus* in Lake Biwa and the surrounding rivers.**

| Lake Biwa | n | Ar (5.42 ± 0.16) | Ho (0.51 ± 0.03) | He (0.50 ± 0.02) |
| --- | --- | --- | --- | --- |
| L1 | 26 | 5.46 | 0.5 | 0.56 |
| L2 | 28 | 5.52 | 0.52 | 0.58 |
| L3 | 28 | 5.33 | 0.45 | 0.55 |
| L4 | 21 | 5.43 | 0.5 | 0.58 |
| L5 | 26 | 5.26 | 0.49 | 0.54 |
| L6 | 22 | 5.33 | 0.52 | 0.54 |
| L7 | 20 | 5.76 | 0.53 | 0.58 |
| L8 | 12 | 5.23 | 0.51 | 0.56 |
| L9 | 20 | 5.48 | 0.53 | 0.57 |
| Rivers | n | Ar (4.47 ± 0.39) | Ho (0.50 ± 0.04) | He (0.55 ± 0.04) |
| R1 | 20 | 4.23 | 0.46 | 0.52 |
| R2 | 21 | 4.68 | 0.45 | 0.48 |
| R3 | 16 | 3.83 | 0.46 | 0.52 |
| R4 | 18 | 5.09 | 0.58 | 0.6 |
| R5 | 10 | 4.57 | 0.53 | 0.59 |
| R6 | 26 | 4.63 | 0.5 | 0.56 |
| R7 | 11 | 4.82 | 0.5 | 0.56 |
| R8 | 10 | 4.29 | 0.53 | 0.56 |
| R9 | 10 | 4.14 | 0.5 | 0.58 |

Lake samples showed significantly higher values of allelic richness than river samples (Mann-Whitney U test, p < 0.001). The mean Ho and He did not show significant differences between lake samples and river samples (Mann-Whitney U test, Ho, p = 0.75; He, p = 0.81). n = sample size, Ar = allelic richness (based on minimum sample size of 10 diploid individuals), Ho = observed heterozygosity, He = expected heterozygosity. The averages and standard deviations of Ar, Ho, and He are shown in the parentheses.
