# Supplementary material for: Morphological variation associated with trophic niche expansion within a lake population of a benthic fish
Source: PLoS One. 2020 Apr 23;15(4):e0232114. doi: 10.1371/journal.pone.0232114 (PMC7179883; doi:10.1371/journal.pone.0232114)

**S1 Fig. Histograms of PC2 and PC3 of local samples of *Pseudogobio esocinus* in Lake Biwa.** Distributions of PC2 (left) and PC3 (right) scores showed unimodality in all the local samples (Silverman’s tests, PC2: L1, p = 0.51; L2, p = 0.83; L3, p = 0.65; L4, p = 0.91; L5, p = 0.64; L6, p = 0.94, and PC3: L1, p = 0.97; L2, p = 0.63; L3, p = 0.65; L4, p = 0.58; L5, p = 0.76; L6, p = 0.69). For sample codes, see Figure 1 and Table 1.


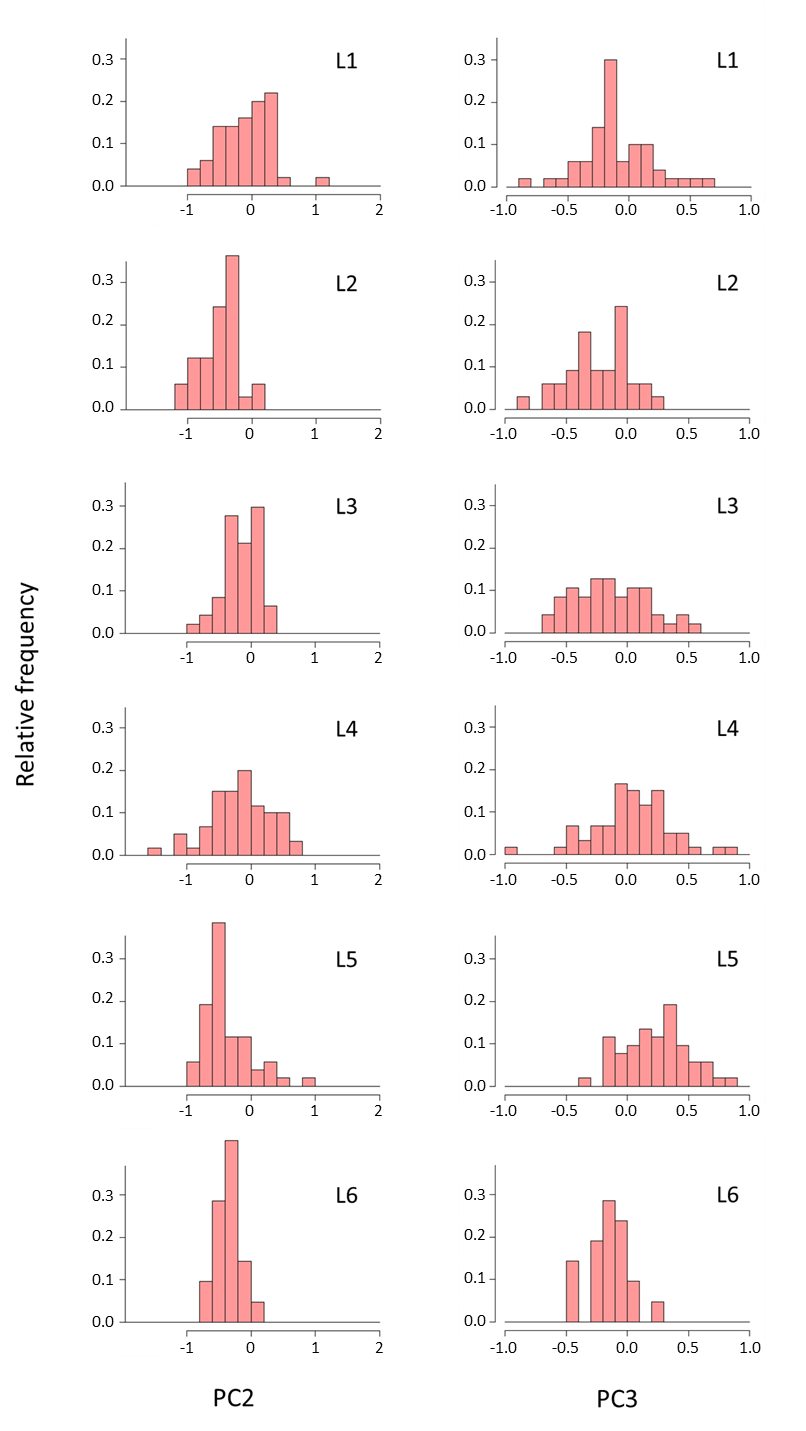

Supplement: S1 Fig — Distributions of PC2 (left) and PC3 (right) scores showed unimodality in all the local samples (Silverman’s tests, PC2: L1, p = 0.51; L2, p = 0.83; L3, p = 0.65; L4, p = 0.91; L5, p = 0.64; L6, p = 0.94, and PC3: L1, p = 0.97; L2, p = 0.63; L3, p = 0.65; L4, p = 0.58; L5, p = 0.76; L6, p = 0.69). For sample codes, see Fig 1 and Table 1. (DOCX) [file pone.0232114.s006.docx]
