# Supplementary material for: Morphological variation associated with trophic niche expansion within a lake population of a benthic fish
Source: PLoS One. 2020 Apr 23;15(4):e0232114. doi: 10.1371/journal.pone.0232114 (PMC7179883; doi:10.1371/journal.pone.0232114)

**S2 Fig. Histograms of PC2 and PC3 of local samples of *Pseudogobio esocinus* in rivers.** Distributions of PC2 (left) and PC3 (right) scores showed unimodality in all the local samples (Silverman’s tests, PC2: R1, p = 0.76; R2, p = 0.83; R3, p = 0.86; R4, p = 0.86; R5, p = 0.61; R6, p = 0.83; R7, p = 0.61, and PC3: R1, p = 0.84; R2, p = 0.79; R3, p = 0.38; R4, p = 0.94; R5, p = 0.7; R6, p = 0.33; R7, p = 0.57). For sample codes, see Figure 1 and Table 1.


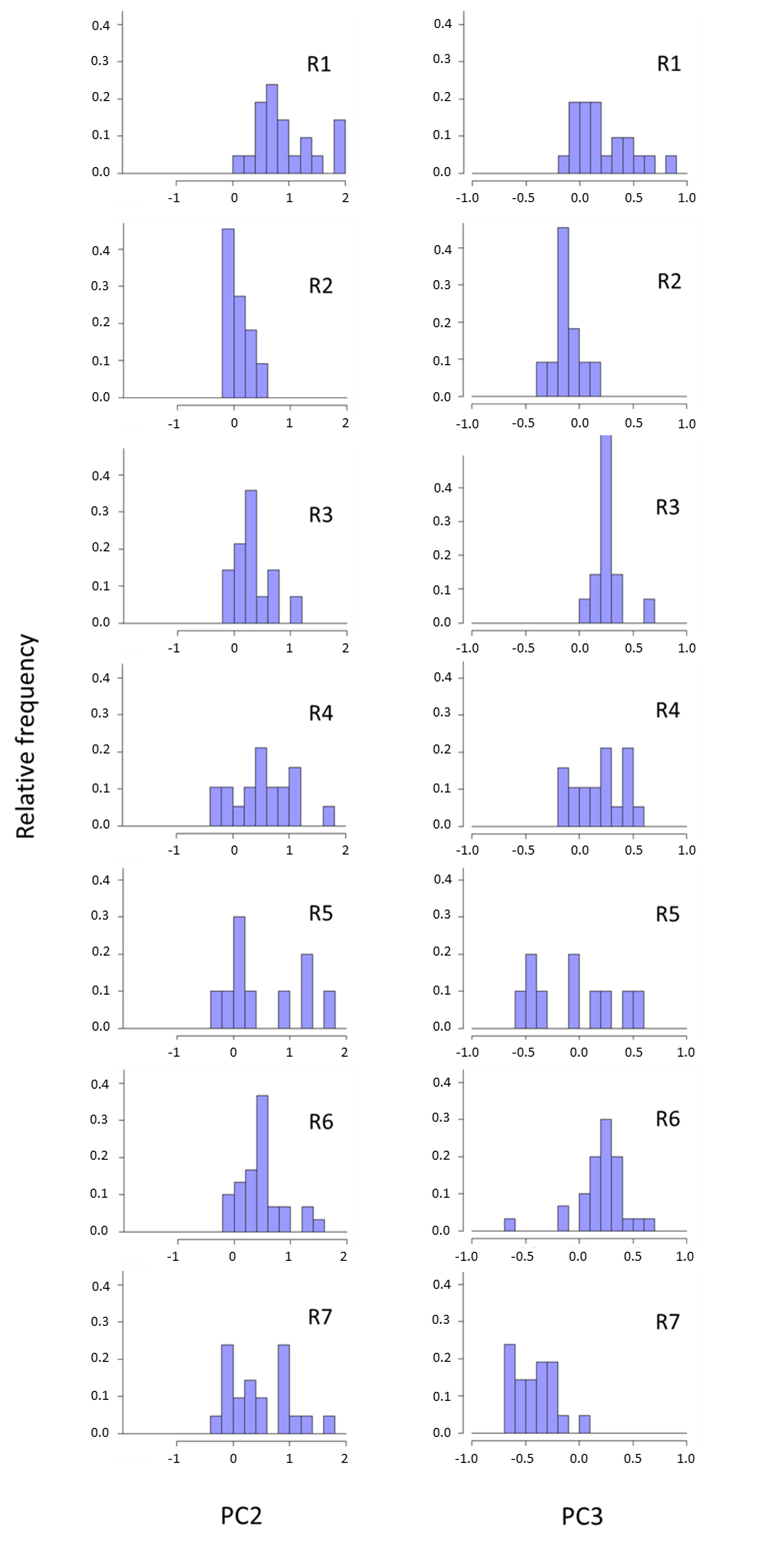

Supplement: S2 Fig — Distributions of PC2 (left) and PC3 (right) scores showed unimodality in all the local samples (Silverman’s tests, PC2: R1, p = 0.76; R2, p = 0.83; R3, p = 0.86; R4, p = 0.86; R5, p = 0.61; R6, p = 0.83; R7, p = 0.61, and PC3: R1, p = 0.84; R2, p = 0.79; R3, p = 0.38; R4, p = 0.94; R5, p = 0.7; R6, p = 0.33; R7, p = 0.57). For sample codes, see Fig 1 and Table 1. (DOCX) [file pone.0232114.s007.docx]
