# Supplementary material for: Morphological variation associated with trophic niche expansion within a lake population of a benthic fish
Source: PLoS One. 2020 Apr 23;15(4):e0232114. doi: 10.1371/journal.pone.0232114 (PMC7179883; doi:10.1371/journal.pone.0232114)

**S3 Fig. Boxplots of PC2, PC3, and PV*i* of local samples of *Pseudogobio esocinus*.** These boxplots show the median (dark horizontal line), 25% and 75% quartiles (the box), and the entire range (the whiskers). Distinct letters indicate significant differences in the scores in multiple comparison test under Bonferroni correction (significance level = 0.05). For sample codes, see Figure 1 and Table 1.


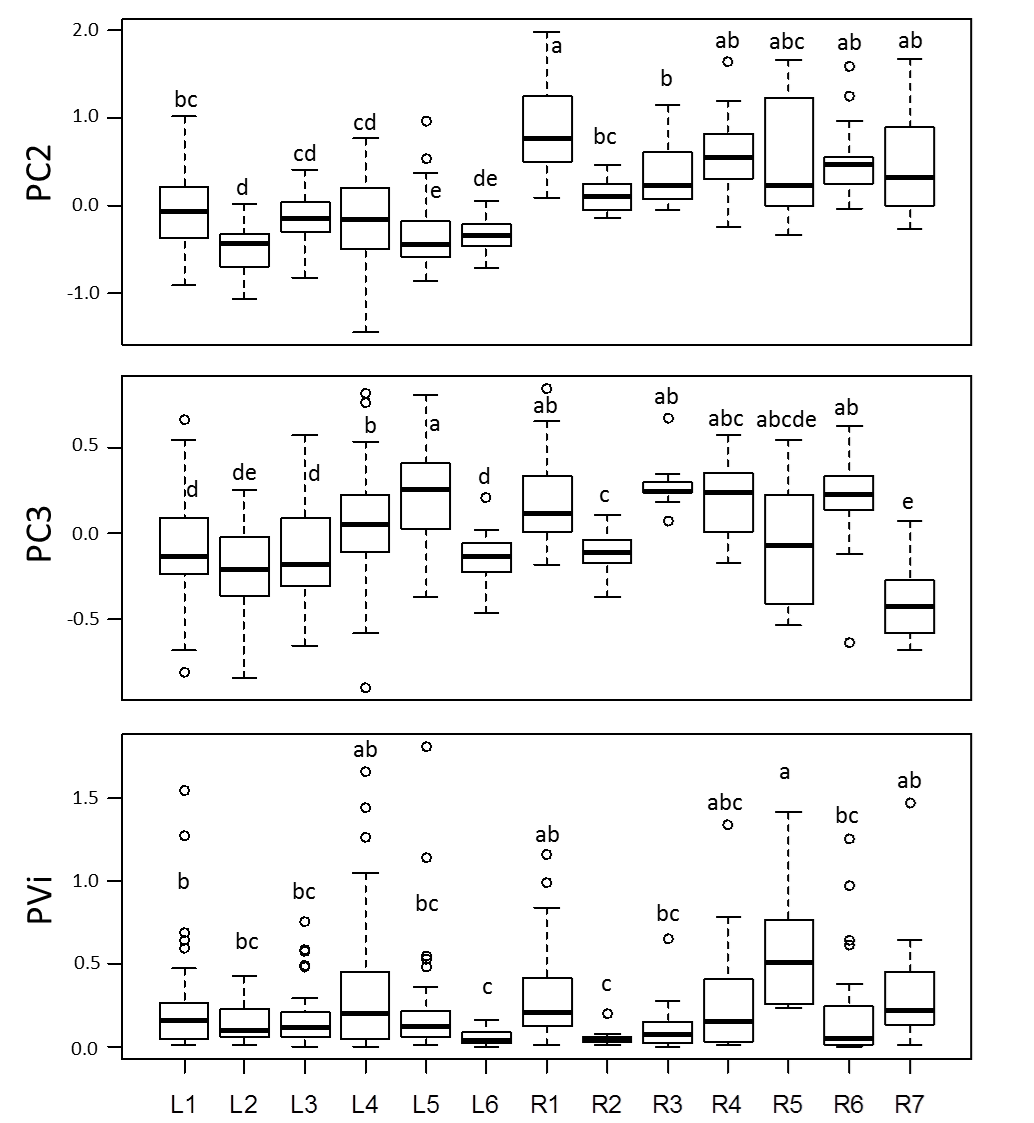

Supplement: S3 Fig — These boxplots show the median (dark horizontal line), 25% and 75% quartiles (the box), and the entire range (the whiskers). Distinct letters indicate significant differences in the scores in multiple comparison test under Bonferroni correction (significance level = 0.05). For sample codes, see Fig 1 and Table 1. (DOCX) [file pone.0232114.s008.docx]
