# Supplementary material for: Morphological variation associated with trophic niche expansion within a lake population of a benthic fish
Source: PLoS One. 2020 Apr 23;15(4):e0232114. doi: 10.1371/journal.pone.0232114 (PMC7179883; doi:10.1371/journal.pone.0232114)

**S4 Fig. Diet compositions of *Pseudogobio esocinus.*** Each specimen was collected from L4 (upper) and L6 (lower) in Lake Biwa.


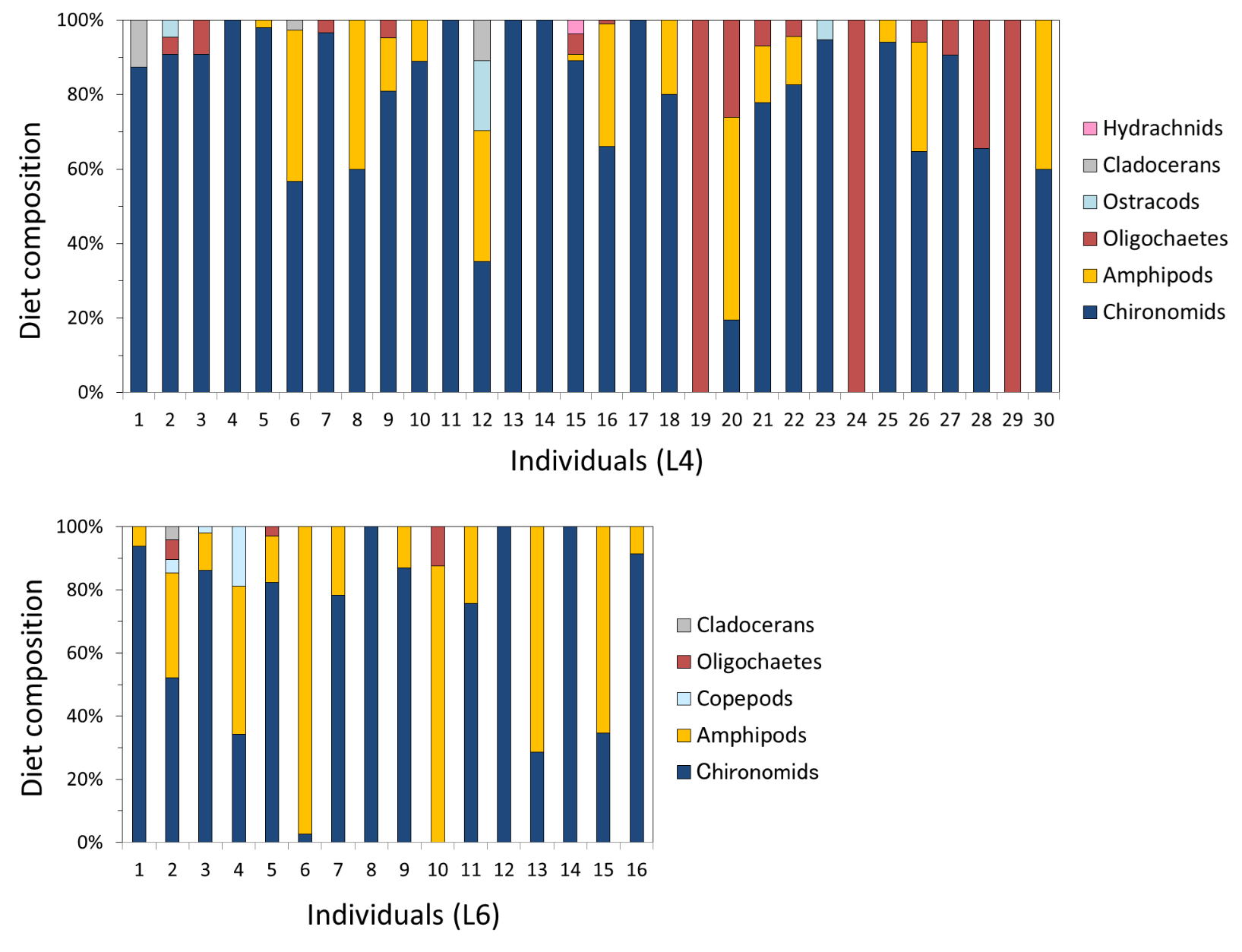

Supplement: S4 Fig — Each specimen was collected from L4 (upper) and L6 (lower) in Lake Biwa. (DOCX) [file pone.0232114.s009.docx]
