# Supplementary material for: Morphological variation associated with trophic niche expansion within a lake population of a benthic fish
Source: PLoS One. 2020 Apr 23;15(4):e0232114. doi: 10.1371/journal.pone.0232114 (PMC7179883; doi:10.1371/journal.pone.0232114)

**S5 Fig. Relationship between phenotypic distances (PC2, PC3, and the ED) and genetic distance of individuals.** All the relationships were not significant (PC2, Mantel statistic r = -0.014, P = 0.65; PC3, Mantel statistic r = -0.031, P = 0.85; ED, Mantel statistic r = -0.035, P = 0.86).


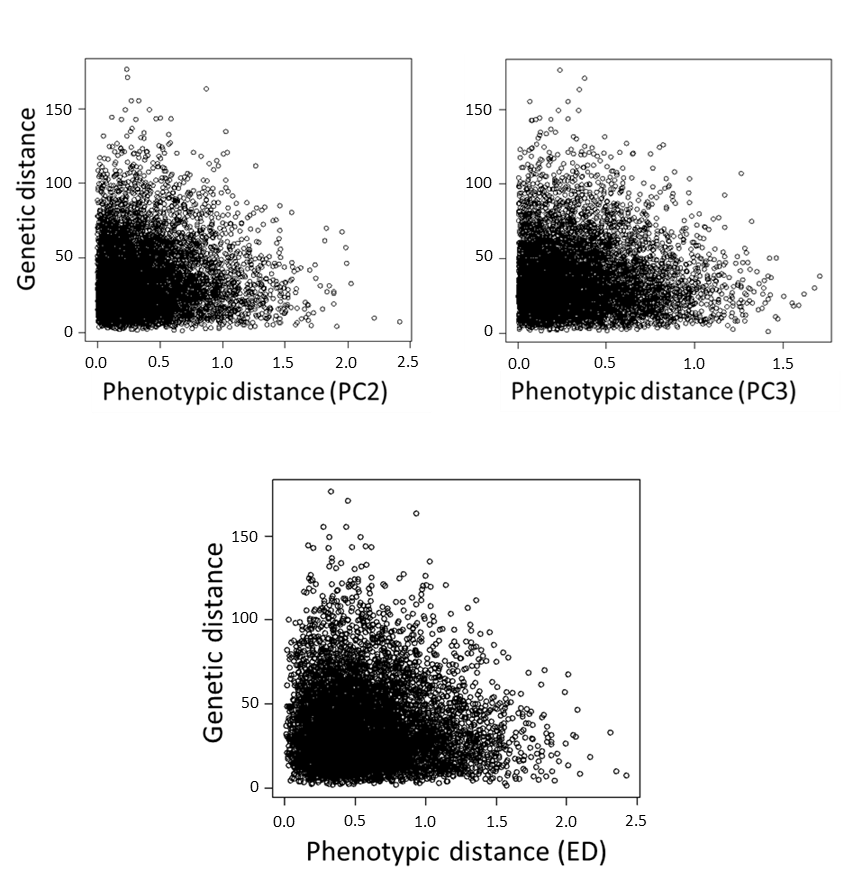

Supplement: S5 Fig — All the relationships were not significant (PC2, Mantel statistic r = -0.014, P = 0.65; PC3, Mantel statistic r = -0.031, P = 0.85; ED, Mantel statistic r = -0.035, P = 0.86). (DOCX) [file pone.0232114.s010.docx]
